# Study of shear properties and shear model of soil-rock mixture

**DOI:** 10.1371/journal.pone.0292384

**Published:** 2023-12-13

**Authors:** Changbo Du, Han Tao, Fu Yi, Xingtao Meng, Di Sun

**Affiliations:** 1 College of Civil Engineering, Liaoning Technical University, Fuxin, China; 2 Beijing Jingneng Geological Engineering Co., Ltd, Beijing, China; 3 SCEGC Mechanized Construction Group Co., Ltd, Xian, China; 4 Liaoning Provincial Communication Planning and Design Institute Co., Ltd, Shenyang, China; Mirpur University of Science and Technology, PAKISTAN

## Abstract

This study investigates the shear characteristics of soil-rock mixtures, a critical factor influencing slope stability in engineering construction. Soil-rock mixtures, often exhibit poor integrity and can easily soften in water due to geological influences. The YT1200 direct shear drawing friction system was employed to conduct shear tests, analyzing the effect of varying water content and fine particle mass fraction under different normal stresses. Utilizing fractional derivatives, we formulated a fractional derivative shear model. Test results illustrated a softening phenomenon post achieving peak shear stress in the soil-rock mixture. It was found that peak shear stress is directly proportional to the normal stress, and inversely proportional to both water content and fine particle mass fraction. Additionally, the cohesion and internal friction angle decrease according to a power function with increasing water content, and non-linearly decrease with the rise of fine particle mass fraction. The proposed shear model aptly simulates the entire shear failure process of the soil-rock mixture, effectively analyzing the influence of key factors on shear characteristics. These findings contribute to the strength prediction and numerical simulation of soil-rock mixtures, thereby aiding in the design of reinforcement schemes and slope stability analysis.

## Introduction

Soil-rock mixture represents a unique geological material that exhibits distinct strength characteristics, residing between soil and rock mass. This complexity often leads to various geological disasters [1], given the soil-rock mixture’s vulnerability to geological structures, poor integrity, and tendency to soften in water. These properties significantly impact the overall slope stability. To better understand the shear performance of soil-rock mixture, numerous studies have been conducted:

Early field research primarily involved in-situ tests. Xu et al. [2] performed horizontal direct shear tests on soil-rock mixtures under natural and soaking conditions. Through compaction and in-situ direct shear tests, Zhang et al. [3] discovered that the presence of rock blocks enhanced the deformation modulus and internal friction angle of soil-rock mixture compared to that of soil samples, albeit at the cost of reduced cohesion. Wei et al. [4] evaluated the high-energy dynamic compaction performance of soil-rock mixture based on field testing. Beyond the field tests, studies have also focused on physical and mechanical properties. For instance, Xu et al. [5] employed digital image processing, a novel approach, to quantitatively discern the proportion and distribution of rock blocks within soil-rock materials. Building upon the fundamentals of linear elastic fracture mechanics of rock and the maximum shear stress criterion, Haeri et al. [6] examined the propagation and direction of microcracks in rock under the influence of a disc cutter. A novel method was then proposed for determining the fracture toughness of rock-like specimens through radial compression tests using the central crack horseshoe disk method [7]. Zhang et al. [8] conducted triaxial tests on soil-rock mixtures, delving into the impact of particle size distribution on the mechanical properties of soil-rock mixtures. Meanwhile, Zhao et al. [9] investigated the correlation between macroscopic deformation, strength, content, size, and random position of rock using medium-scale shear and triaxial tests. Further, Xu et al. [10] revealed that the deviatoric stress and friction strength of soil-rock mixture increased proportionally with rock content. The relationship between the shear strength of soil-rock mixtures and rock block proportion was demonstrated by Zhang et al. [11] to be nonlinear. An improved theoretical composite seepage model for the permeability coefficient of soil-rock mixture and the relative content of each component (pure soil and pure broken rock) was proposed by Zhou et al. [12]. In the realm of numerical simulation, increased scholarly attention is being given to soil-rock mixture studies. For instance, Shan et al. [13] introduced an automatic generation method of a PFC two-dimensional numerical model of soil-rock mixture microstructure, based on digital image processing. For instance, Yang et al. [14] employed the numerical manifold method to assess soil-rock mixture’s slope stability, while Li et al. [15] combined numerical simulation and CT scanning triaxial tests to analyze the properties of soil-rock mixture. Sarfarazi et al. [16] utilized the particle flow code to simulate and identify the mode I fracture toughness of rock through direct and indirect methods. Ji et al. [17] leveraged the discrete element method and CT scanning to establish a numerical simulation method for the vibration compaction method of soil- rock mixtures, specifically those with a maximum particle size exceeding 40 mm. Furthering the applications of the discrete element method, Xu et al. [18] carried out biaxial test, revealing nonlinear characteristics in the shear strength of soil-rock mixture. Duncan et al. [19] explored the impact of stone content on the sliding surface of soil-rock mixture slopes based on the strength reduction method. Through a blend of testing and numerical simulation, Hu et al. [20] researched the strength, deformation, and permeability characteristics of soil-rock mixture and its structural control mechanisms. Zhao et al. [21] proposed a novel approach for constructing digital models of soil-rock mixture, allowing for the creation of soil-rock mixture with different block size distributions and stone content. While the aforementioned research primarily focuses on the effects of stone content or particle size on the shear characteristics of soil-rock mixture, factors like water content and fine particle mass fraction also exert significant influence on these characteristics. As such, these areas require further investigation.

In the context of soil-rock mixture shear models, the curve of shear stress against shear displacement is a fundamental resource for studying material shear characteristics, as it reveals the stress and deformation behavior of the material. As such, this has been a focal point of various scholarly research efforts. Wang et al. [22] discovered an approximate linear relationship between the maximum shear modulus and the rock content, alongside an exponential relationship between the maximum shear modulus and the normal stress. An extended Mohr-Coulomb criterion in the form of a power function to represent the strength envelope was proposed by Zhang et al. [23]. Xu et al. [24] established a power function model describing the shear deformation constitutive relationship of weak interlayers, based on field shear test data. Zhou et al. [25] examined the macroscopic damage model of soil-rock mixture, applying PFC3D to scrutinize the impact of strength damage between soil and rock particles under freeze-thaw cycles. Yao et al. [26] employed the Weibull cumulative distribution and Gaussian density function for quantitatively evaluating the influence of particle shape. Meanwhile, Venkateswarlu et al. [27] analyzed the applicability of the Barton and Kjaernsli shear strength models. While a plethora of research on soil-rock mixture shear models has been undertaken, few models effectively reflect strain softening, and improvements in model accuracy are needed.

In conclusion, this study employed large-scale direct shear tests to explore the shear characteristics and shear models of soil-rock mixtures. These tests yielded the shear stress-shear displacement curves of soil-rock mixtures under normal stress, accounting for various water contents and fine particle mass fractions. The paper provides an in-depth analysis of how normal stress, water content, and particle characteristics impact the shear strength and shear strength index of soil-rock mixtures. By harnessing the collected experimental data, a fractional derivative shear model of soil-rock mixtures–in other words, a constructive shear model–was derived. This model offers a theoretical foundation for the prediction of soil-rock mixture strength and its numerical simulation. Furthermore, it serves as a basis for the analysis of slope stability and the design of reinforcement schemes for soil-rock mixture.

## Direct shear test of soil-rock mixture

### Test material

The material for this study was sourced from the weak interlayer section of an open-pit iron mine in Liaoning. Field investigations revealed that soil-rock mixtures constitute the primary component of these weak interlayers. The filler between particles tends to soften when exposed to water, and a portion of it becomes gravel soil after disturbance, making it challenging to obtain original samples. Ultimately, several sets of samples were chiseled from the surfaces of three distinct weak interlayers using a geological hammer. To mitigate sample disturbance and water loss during transportation, each sample was carefully wrapped and secured in multiple layers of preservative film. Upon arrival at the laboratory, the soil-rock mixture was dried, revealing a natural moisture of 8.4%. The dried sample was then crushed with a hammer and sifted using a standard sieve apparatus, yielding an average mass fraction of fine particles at 17.12%.

During the field sampling, a wide particle size distribution was observed in the soil-rock mixture. To investigate the shear performance of the soil-rock mixture across different particle gradations, it becomes crucial to eliminate the impact of water content and particle cementation on the mixture’s shear performance. Thus, remolded samples with different gradations were designed for comparative testing. In the design of remolded samples, the mass fraction of fine particles in the natural soil-rock mixture was used as a reference. Following this, three sample groups were designed to proportionally increase or decrease from this reference. As a result, a total of four sample groups, including the original sample, were established, with fine particle mass fractions of 8.56%, 17.12% (original sample), 25.68%, and 34.24%, respectively.

**Fig 1:**
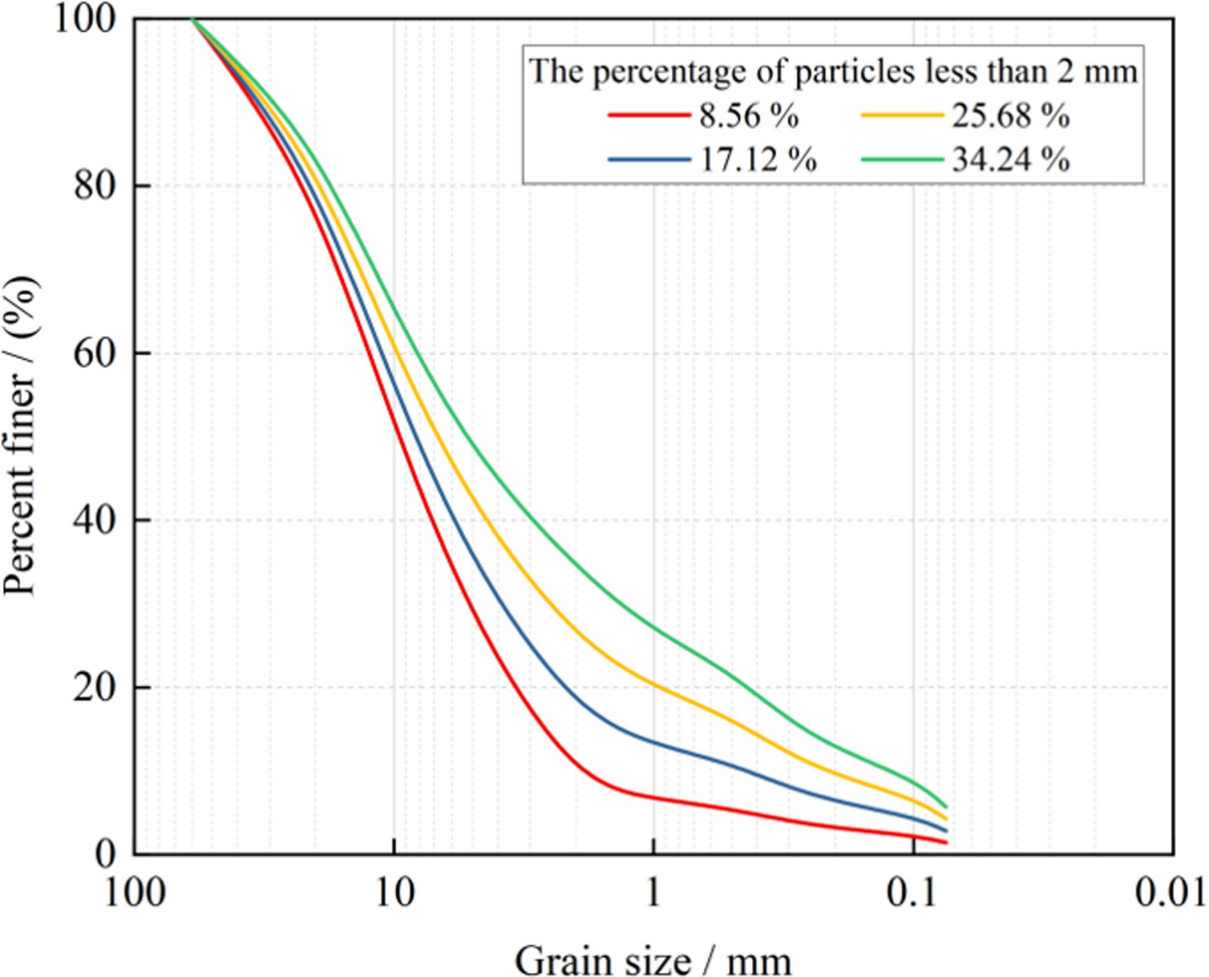
Grading curve.

**Fig 2:**
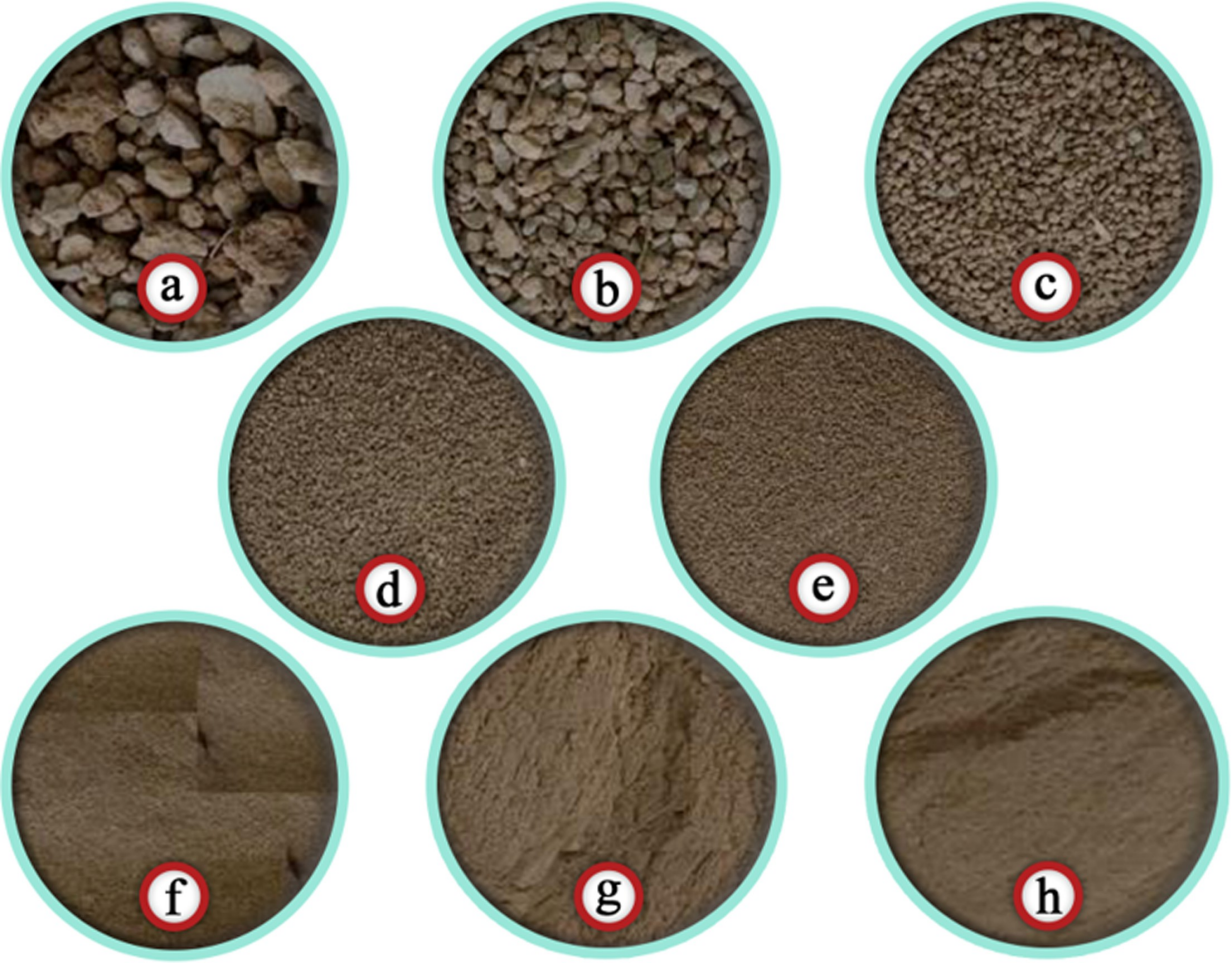
Sample after screening.

The grading curves of the four sample groups are depicted in Fig 1, while Fig 2 shows the samples post-screening. The grading curves reveal a pattern of an initial steep decline, followed by a gradual descent, creating an overall concave shape. This pattern indicates a significantly higher mass fraction of coarse particles in the weak interlayer compared to fine particles, suggesting the weak interlayer primarily comprises coarse particles. For geotechnical materials of low strength, the non-uniformity coefficient *C*_*u*_ and the curvature coefficient *C*_*c*_ of the particle gradation curve are typically used to describe the engineering properties of the materials. The grading analysis results for the four sample groups are presented in Table 1. It is generally considered that when the conditions *C*_*u*_ > 5 and *C*_*c*_ = 1 ∼ 3 are satisfied, the material exhibits good gradation, high strength, and superior engineering properties. The original samples used in this study meet the conditions *C*_*u*_ = 26.28 > 5 and *C*_*c*_ = 2.70, indicating good gradation. The original sample *C*_*u*_ value, which is much higher than 5, suggests less space exists between the particles of the soil-rock mixture, and the inter-filling among the particles results in higher density. The remolded samples 1, 3, and 4 also meet the conditions *C*_*u*_ > 5 and *C*_*c*_ = 1 ∼ 3, indicating good gradation as well.

**Table 1:** Sample particle size distribution analysis results.

Sample group number	Mass fraction of fine particles/(%)	*d*_10_(mm)	*d*_30_(mm)	*d*_60_(mm)	*C*_*u*_	*C*_*c*_
Sample 1 (remolded)	8.56	2.12	5.28	12.43	5.86	1.06
Sample 2 (original)	17.12	0.43	3.62	11.30	26.28	2.70
Sample 3 (remolded)	25.68	0.22	2.52	9.79	44.50	2.95
Sample 4 (remolded)	34.24	0.12	1.36	8.11	67.58	1.90

### Test equipment

**Fig 3:**
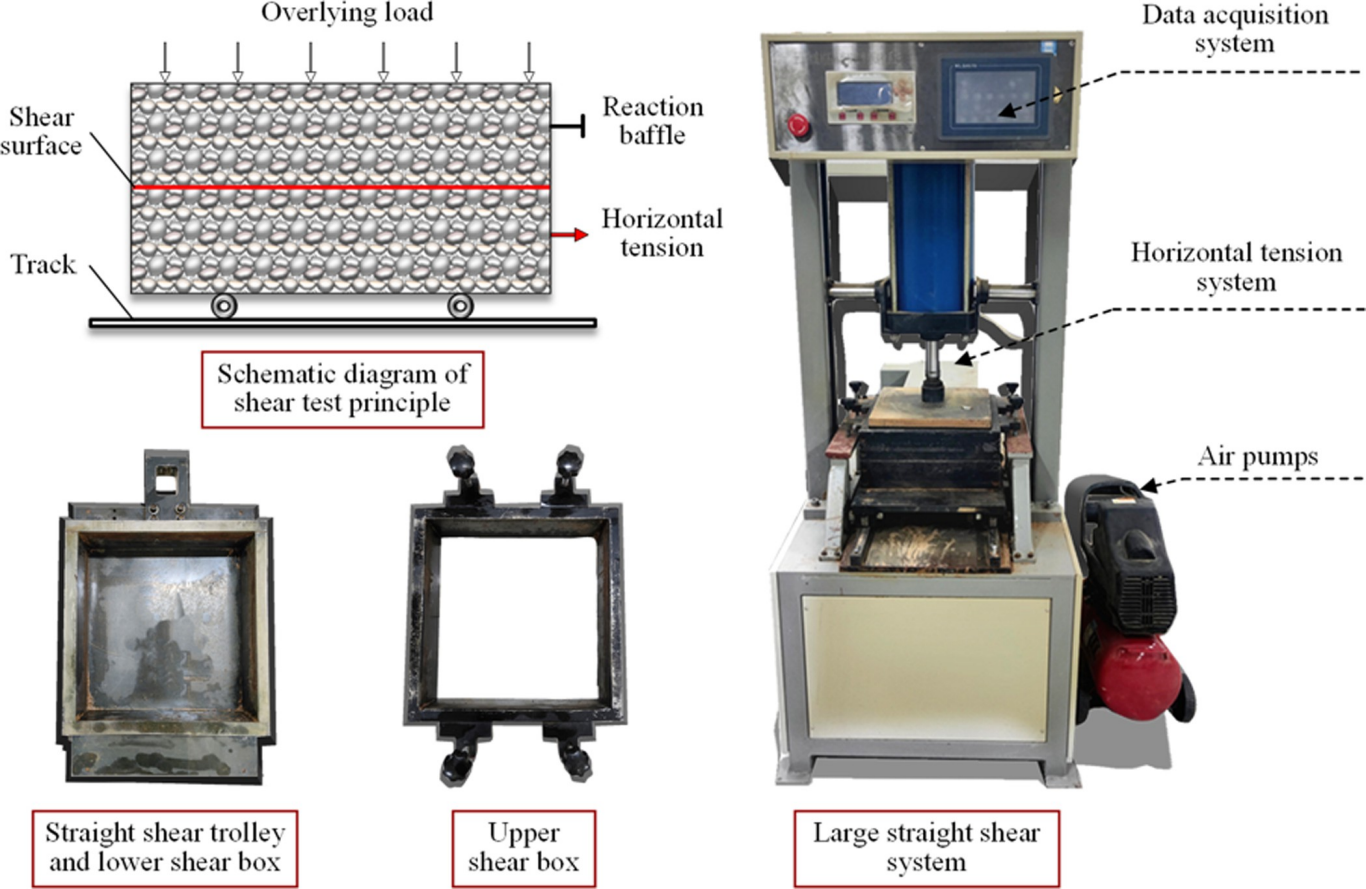
Large direct shear test system.

Given the difficulty in accurately measuring the shear strength of the soil-rock mixture using the common small direct shear equipment, an automatic large-scale direct shear test system was employed for this experiment. The system consists of four primary components: a vertical pressure controller, a horizontal tension system, a data acquisition system, and a test box, as depicted in Fig 3. The vertical pressure controller operates via air pressure loading (an external air pressure pump is required), with a 30 × 30 cm pressure plate installed underneath the cylinder piston. The horizontal pulling force was supplied by a ball screw system, a precision linear module. The range of the drawing rate ranged from 0 to 5 mm/min, and the drawing displacement could reach up to 100 mm. The data acquisition system was designed to display the test parameters (including shear rate, normal pressure, drawing rate, etc.) and results (like the shear displacement-stress curve) on an electronic screen. Furthermore, to mitigate size effect, the custom-made shear box was divided into two parts: upper and lower. The internal dimensions were 300 mm × 300 mm × 75 mm (length × width × height). The lower shear box was fixed onto the direct shear carriage, while the height of the upper shear box could be independently adjusted. This setup helps avoid frictional effects on the accuracy of test results during the shearing process.

### Test scheme

In this study, two experimental schemes were designed. The first aimed to investigate the impact of water content on the shear characteristics of the soil-rock mixture, while the second sought to analyze the effect of particle characteristics on these shear characteristics. In the first scheme, we aimed to isolate the effect of water content by using the natural water content of the soil-rock mixture as a benchmark, increasing and decreasing it proportionally. The four groups of samples had natural water content, levels of 4.2%, 8.4% (original sample), 16.8%, and 33.6%, respectively, all maintaining a fine particle mass fraction of 17.12%. To achieve the targeted water content, water was injected into the original sample in controlled manner, mixed thoroughly, divided into four layers, and then loaded into the shear box for consolidation and leveling. The second scheme involved the use of fine particle mass fraction of the natural soil-rock mixture as a benchmark. It was used to design three additional groups of samples. The fine particle mass fractions in these four groups were 8.56%, 17.12% (original sample), 25.68%, and 34.24%, respectively, all keeping the water content at the natural level of 8.4%. Considering the depth of the weak interlayer and the loading capacity of the test equipment, four different overburden loads were utilized for shear testing. Each scheme included four groups of samples, making a total of 32 shear tests across the two schemes. The shear rate was set to 0.3 mm/min. Further details of the test scheme are presented in Table 2.

**Table 2:** Experimental program design.

Test scheme	Sample group number	Mass fraction of fine particles/(%)	Water content/(%)	Overburden load pressure/kPa	Remark
The first test scheme	Sample 1 (remolded)	17.12	4.2	50、100、150、200	The influence of water content on the shear characteristics of soil-rock mixture is analyzed.
	Sample 2 (original)		8.4		
	Sample 3 (remolded)		16.8		
	Sample 4 (remolded)		33.6		
The second test scheme	Sample 1 (remolded)	8.56	8.4%		The influence of particle characteristics on the shear characteristics of soil-rock mixture is analyzed.
	Sample 2 (original)	17.12			
	Sample 3 (remolded)	25.68			
	Sample 4 (remolded)	34.24			

## Test result analysis

### Analysis of influence of water content on shear characteristics of soil-rock mixture

#### Characteristics of shear stress-shear displacement curve

**Fig 4:**
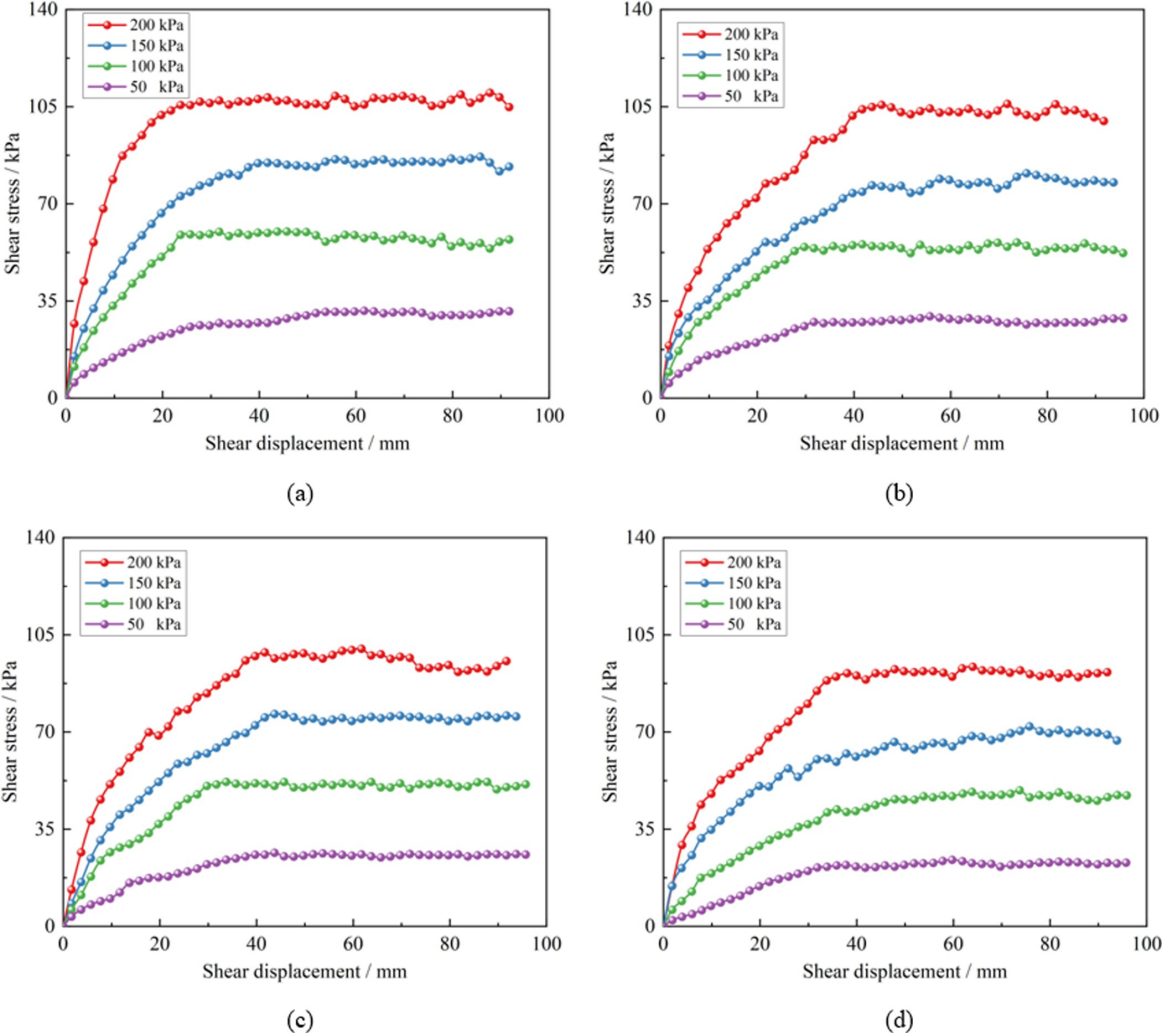
Shear stress-shear displacement curve. (a) Water content 4.2%, (b) Water content 8.4%, (c) Water content 16.8%, (d) Water content 33.6%.

The large-scale shear test system was utilized to conduct shear tests on soil-rock mixture samples with varying water content, and peak shear stress under these different water content conditions was determined as summarized in Table 3. The shear stress-shear displacement curve under different water content is depicted in Fig 4, and several conclusions can be drawn from these results:

- Under the same water content, an increase in normal stress leads to a higher degree of particle interlocking, making it more difficult for the particles to roll and fracture. This results in the appearance of a ’serrated’ curve, and the peak shear stress also rises. The higher the normal stress, the faster the shear stress increases in the initial stage, leading to the faster occurrence of peak stress.
- For soil-rock mixture under the same normal stress, an increase in water content boosts the pore water pressure during shearing, reducing the degree of particle interlocking. The lubricating effect of water on the shear surface becomes more pronounced, which in turn continuously decreases the peak shear stress value. A larger shear displacement is needed to reach the peak shear stress. As water content increases from 4.2% to 33.6%, the peak shear stress corresponding to different overlying loads drops by over 15%. This suggests that water content is a key factor influencing the shear strength of soil-rock mixtures.
- The shear deformation process of the soil-rock mixture, as described by the curve, can be summarized into three stages: elasticity, strain hardening, and strain softening.

**Table 3:** Shear test results of water content characteristics.

Sample group number	Water content/(%)	Shear strength under different overburden pressure/kPa				Fitting formula	*c*/kPa	*φ*/(°)	*R* ^*2*^
		50	100	150	200				
Sample 1 (remolded)	4.2	31.49	60.05	86.99	109.99	*τ* = 0.53*σ*_*n*_ + 6.52	6.52	27.9	0.99
Sample 2 (original)	8.4	29.50	56.08	81.01	106.00	*τ* = 0.51*σ*_*n*_ + 4.54	4.54	27.0	0.99
Sample 3 (remolded)	16.8	26.50	52.05	76.51	100.01	*τ* = 0.49*σ*_*n*_ + 2.52	2.52	26.1	0.99
Sample 4 (remolded)	33.6	23.98	49.00	72.01	93.51	*τ* = 0.46*σ*_*n*_ + 1.73	1.73	24.8	0.99

#### Analysis of shear performance changes

**Fig 5:**
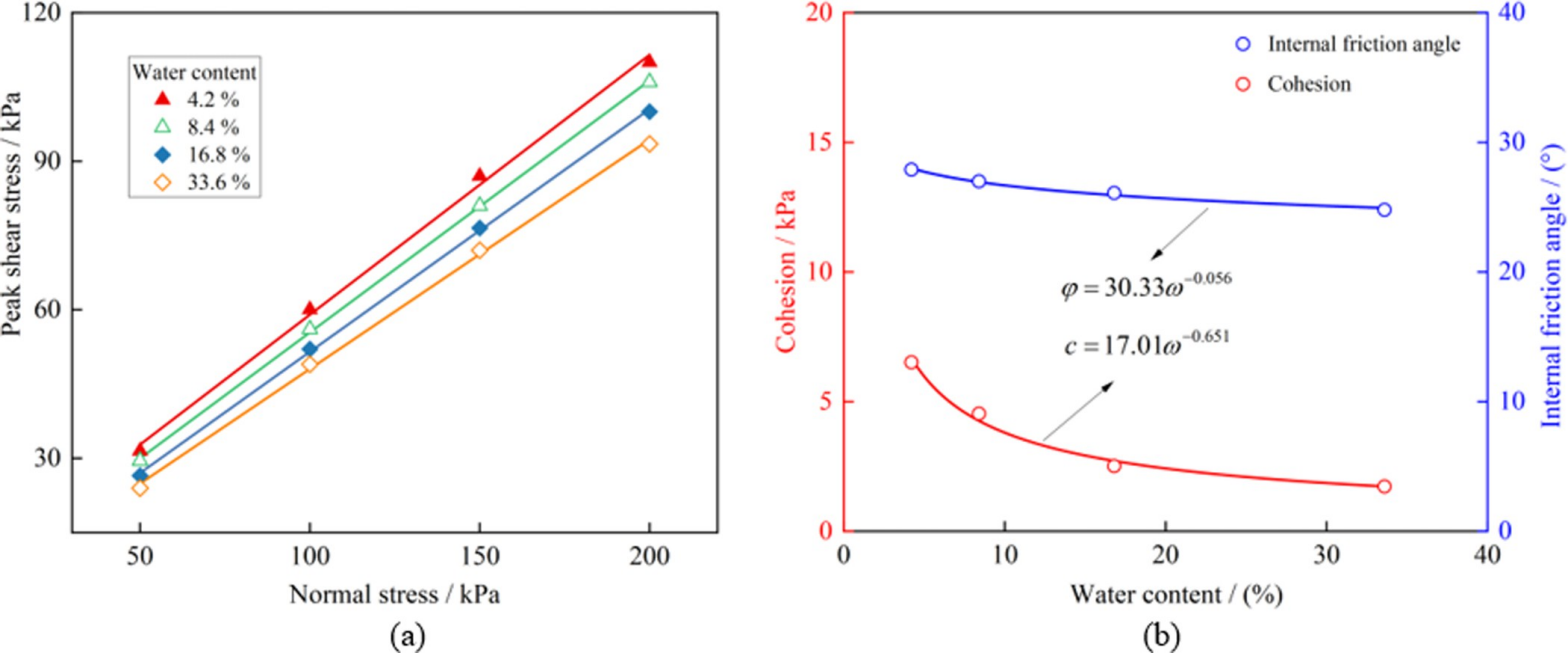
Analysis of shear performance change. (a) Shear strength linear fitting, (b) Change of cohesion and internal friction angle.

The Mohr-Coulomb criterion was used to linearly fit the peak shear strength of samples under varying normal stress to understand the influence of water content on the shear performance of soil-rock mixture. The results of this fitting are presented in Table 3. It is noticeable from the table that the correlation coefficient *R*^2^ is greater than 0.99, indicating a good linear fit. The relationship between the peak shear stress and normal stress for each group is shown in Fig 5(A). When the water content is 33.6%, the cohesion of the soil-rock mixture reaches the lowest value of 1.73 kPa, a reduction of 73.47%. while the internal friction angle decreases from 27.9° to 24.8°, a reduction of 11.11%. The decrease in cohesion of the soil-rock mixture is greater than that of the internal friction angle increases.

Meanwhile, using the 16 sets of shear test results, we can obtain the correlation between *c*, *φ* values and water content *ω*. This correlation is illustrated in Fig 5(B). It was found that there is a power function relationship between *c*, *φ* values and water content *ω*, which can represented using Eqs (1) and (2):

$$c=17.01\omega^{-0.651}(R^{2}=0.99)$$ (1)

$$\varphi =30.33\omega^{-0.056}(R^{2}=0.99)$$ (2)

The water content in a soil-rock mixture plays a crucial role in defining the cohesion between particles. At lower water contents, the particles primarily involve strongly bound water, leading to higher particle cohesion. However, as the water content increases, the nature of the water associated with particles shifts toward being largely outer bound water. This shift results in a drastic reduction in the gravitational force between particles and the cohesive force of the bound water film, consequently causing a sharp decrease in overall cohesion. Furthermore, higher water content facilitates the lubrication effect of water, reducing the sliding frictional force between particles and thereby lowering the internal friction angle. This suggests that soil-rock mixtures can easily soften in the presence of water.

### Analysis of influence of particle characteristics on shear characteristics of soil-rock mixture

#### Characteristics of shear stress-shear displacement curve

**Fig 6:**
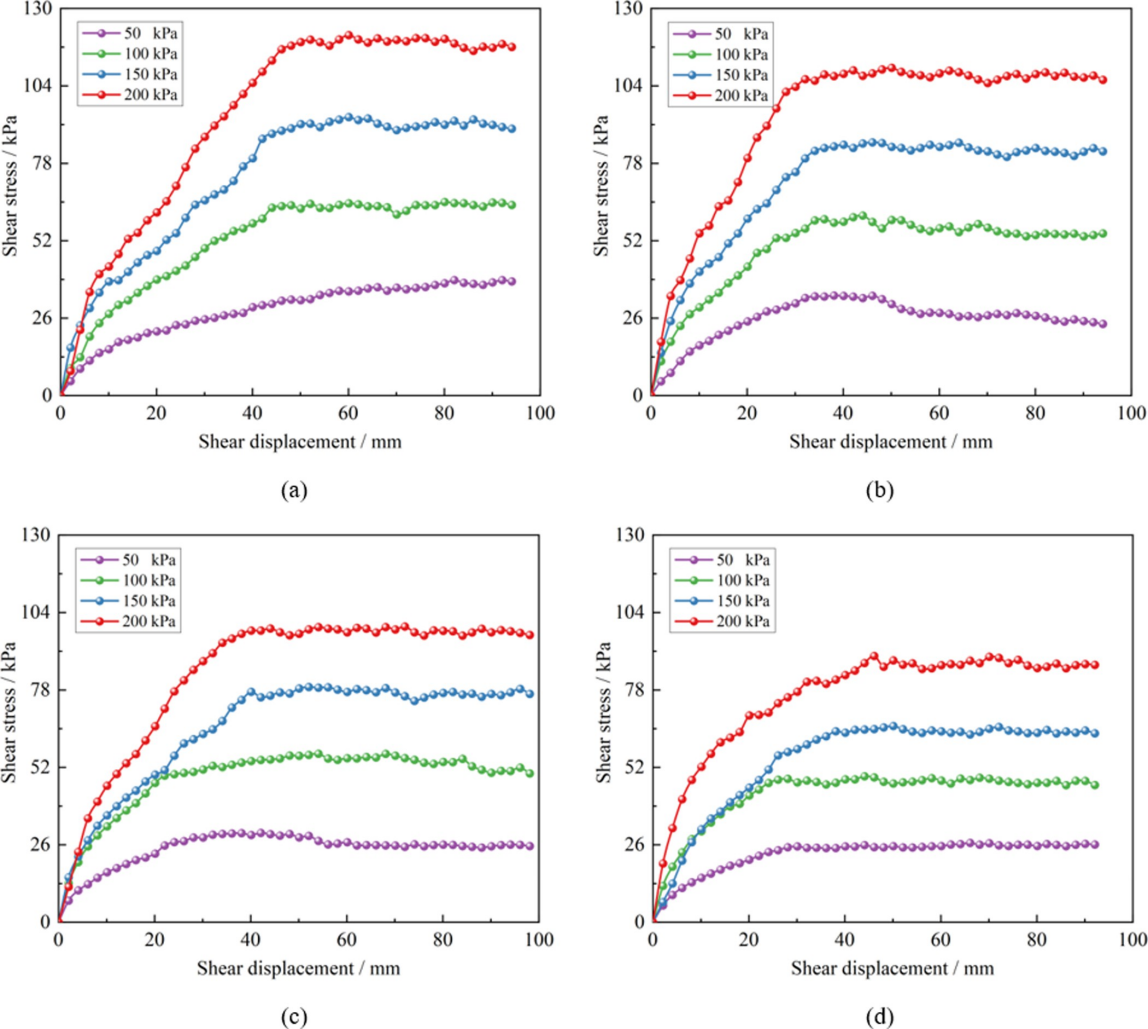
Shear stress-shear displacement curve. (a) Mass fraction of fine particles is 8.56%, (b) Mass fraction of fine particles is 17.12%, (c) Mass fraction of fine particles is 25.68%, (d) Mass fraction of fine particles is 34.24%.

The peak shear stress of the sample under varying overburden pressures was extracted, providing the shear strength of the soil-rock mixture samples under these conditions, as displayed in Table 4. The shear stress-shear displacement curves of the specimens under differing overburden pressures are depicted in Fig 6. The graph shows that the shape of direct shear test curves across different fine particle mass fractions remains relatively consistent. In the initial phase, the shear stress-shear displacement curve grows approximately linearly, with the soil-rock mixture in an elastic state. In the middle phase, the shear stress increase is nonlinear, indicating plastic deformation in soil-rock mixture. Finally, in the failure phase, the shear stress stabilizes or undergoes strain softening as shear displacement increases. As the fine particle mass fraction increases, the rate of shear stress growth initially rises, then falls, and finally increases again. Specifically, the rate of shear stress growth is the highest when the mass fraction of fine particles is 17.12%. Under the same fine particle mass fraction, a higher overburden pressure results in a greater density of the soil-rock mixture and correspondingly, a higher peak stress. Conversely, under the overburden pressure, higher fine particle mass fraction correlate with weaker overall shear capacity of the soil-rock mixture.

**Table 4:** Particle characteristic shear test results.

Sample group number	Mass fraction of fine particles/(%)	Shear strength under different overburden pressure/kPa				Fitting formula	*c*/kPa	*φ*/(°)	*R* ^2^
		50	100	150	200				
Sample 1 (remolded)	8.56	38.78	65.00	93.50	121.01	*τ* = 0.55*σ*_*n*_ + 10.78	10.78	28.8	0.99
Sample 2 (original)	17.12	33.60	60.45	85.00	110.06	*τ* = 0.51*σ*_*n*_ + 8.81	8.81	26.9	0.99
Sample 3 (remolded)	25.68	29.98	56.59	78.99	99.29	*τ* = 0.46*σ*_*n*_ + 8.63	8.63	24.7	0.99
Sample 4 (remolded)	34.24	26.65	49.00	65.89	89.37	*τ* = 0.41*σ*_*n*_ + 6.47	6.47	22.3	0.99

#### Analysis of shear performance changes

**Fig 7:**
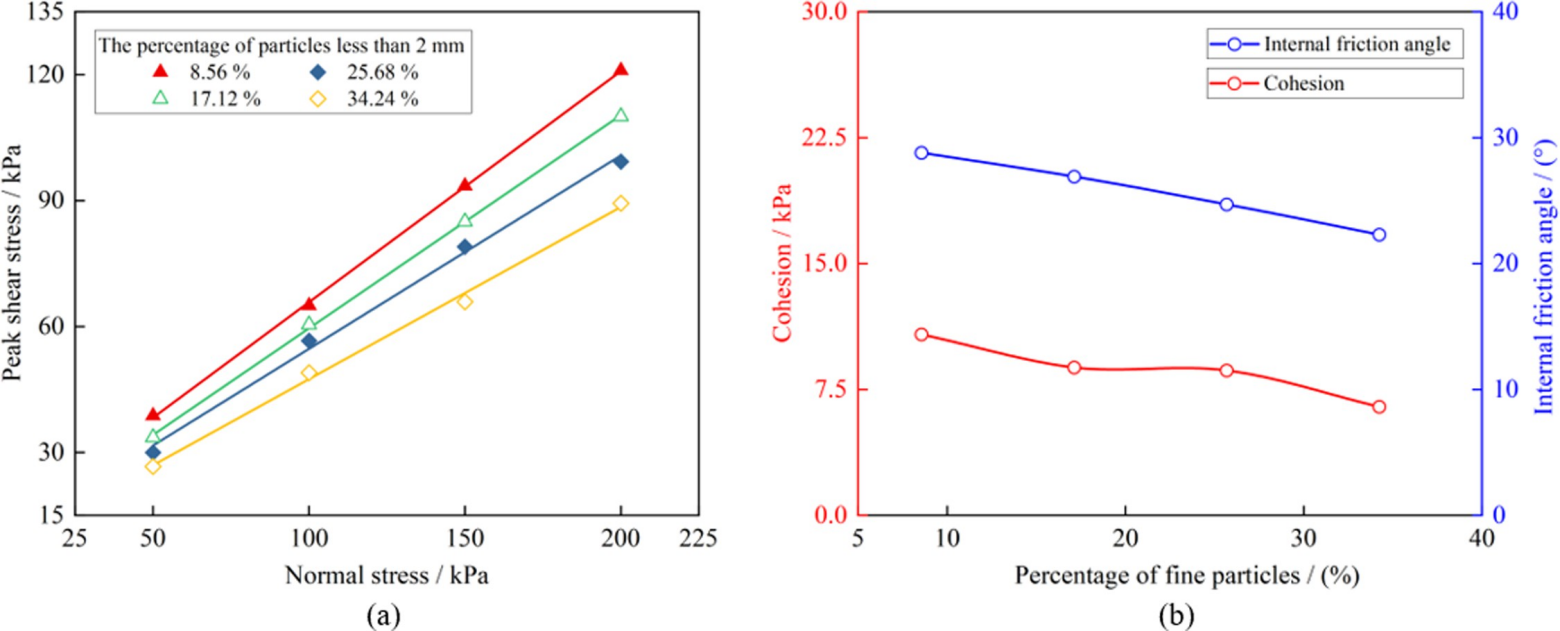
Analysis of shear performance change. (a) Shear strength linear fitting, (b) Change of cohesion and internal friction angle.

To discern the impact of particle characteristics on the shear performance of soil-rock mixture, the Mohr-Coulomb criterion was utilized to fit the peak shear strength of the samples linearly under different normal stress levels. The fitting results, indicated in Table 4, show that the correlation coefficient *R*^2^ is greater than 0.99, suggesting a high degree of linear fit. The relationship between the peak shear stress and normal stress for each group is illustrated in Fig 7(A). When the mass fraction of fine particles rises from 8.56% to 34.24%, the cohesion of the soil-rock mixture drops from 10.78 kPa to 6.47 kPa, a decline of 39.98%, while the internal friction angle reduces from 28.8° to 22.3°, a decrease of 22.57%.

From the 16 sets of shear test results, the correlation between *c*, *φ* values and the fine particle mass fraction can be identified, as shown in Fig 7(B). When the stone content is high, the soil-rock mixture exhibits a skeleton-void structure, with large gaps existing between the block stones. In the initial stage of shearing, under overburden pressure, smaller stones move to the gaps between the larger stones, leading to a densification process in the sample, and thus, the growth rate of shear stress is not at its maximum. As the mass fraction of fine particles increases, the soil-rock mixture transitions to a skeleton-dense structure, resulting in higher degrees of occlusion between the blocks, and the growth rate of shear stress gradually reaches its maximum. Once the mass fraction of fine particles surpasses a certain value, the soil-rock mixture assumes a suspended-dense structure, beginning to exhibit the characteristics of fine particles, and the sample will quickly reach peak shear stress. In these tests, the mass fraction of fine particles is less than 50%, meaning the block stone skeleton of the soil-rock mixture is essentially formed. As the mass fraction of fine particles increases, it essentially represents a similar filling effect as soil, leading to decreases in both cohesion and the internal friction angle.

## Study on shear model of soil-rock mixture

### Traditional shear model

The presence of soil-rock mixed interlayers in rock slopes can lead to shear failure along the structural plane. Traditional shear models, include exponential and hyperbolic models, have been used to analyze such scenarios.

The exponential shear model is expressed in Eq (3):

$$\tau =a_{1}\cdot \left[1-\exp(-b_{1}\cdot x)\right]$$ (3)

By taking the first derivative of Eq (3), the shear stiffness *K*_1_ of the material at each displacement time point can be calculated as shown in Eq (4):

$$K_{1}=\frac{\text{d}\tau }{\text{d}x}=a_{1}\cdot b_{1}\cdot \exp(-b_{1}\cdot x)$$ (4)

From Eq (3), the peak shear stress *τ*_*m*1_ = *a*_1_ can be derived, while the initial shear stiffness *K*_*s*1_ = *a*_1_⋅*b*_1_ can be derived from Eq (4).

The hyperbolic shear model is given in Eq (5):

$$\tau =x/\left(a_{2}+b_{2}\cdot x\right)$$ (5)

The shear stiffness *K*_2_ at each displacement time point can be calculated by taking the first derivative of Eq (5), as shown in Eq (6):

$$K_{2}=\frac{\text{d}\tau }{\text{d}x}=a_{2}/{\left(a_{2}+b_{2}\cdot x\right)}^{2}$$ (6)

where *τ* represents the shear stress (in kPa), *x* denotes the shear displacement (in mm), *a*_1_, *b*_1_ signify the undetermined parameters, *K*_1_ denotes the shear stiffness of the exponential model (in kPa/mm), *a*_2_, *b*_2_ signify the parameters to be determined, and *K*_2_ represents the shear stiffness of the hyperbolic model (in kPa/mm).

The peak shear stress *τ*_*m*2_ = 1/*b*_2_ can be derived from Eq (5), while the initial shear stiffness *K*_*s*2_ = 1/*a*_2_ can be obtained from Eq (6).

**Fig 8:**
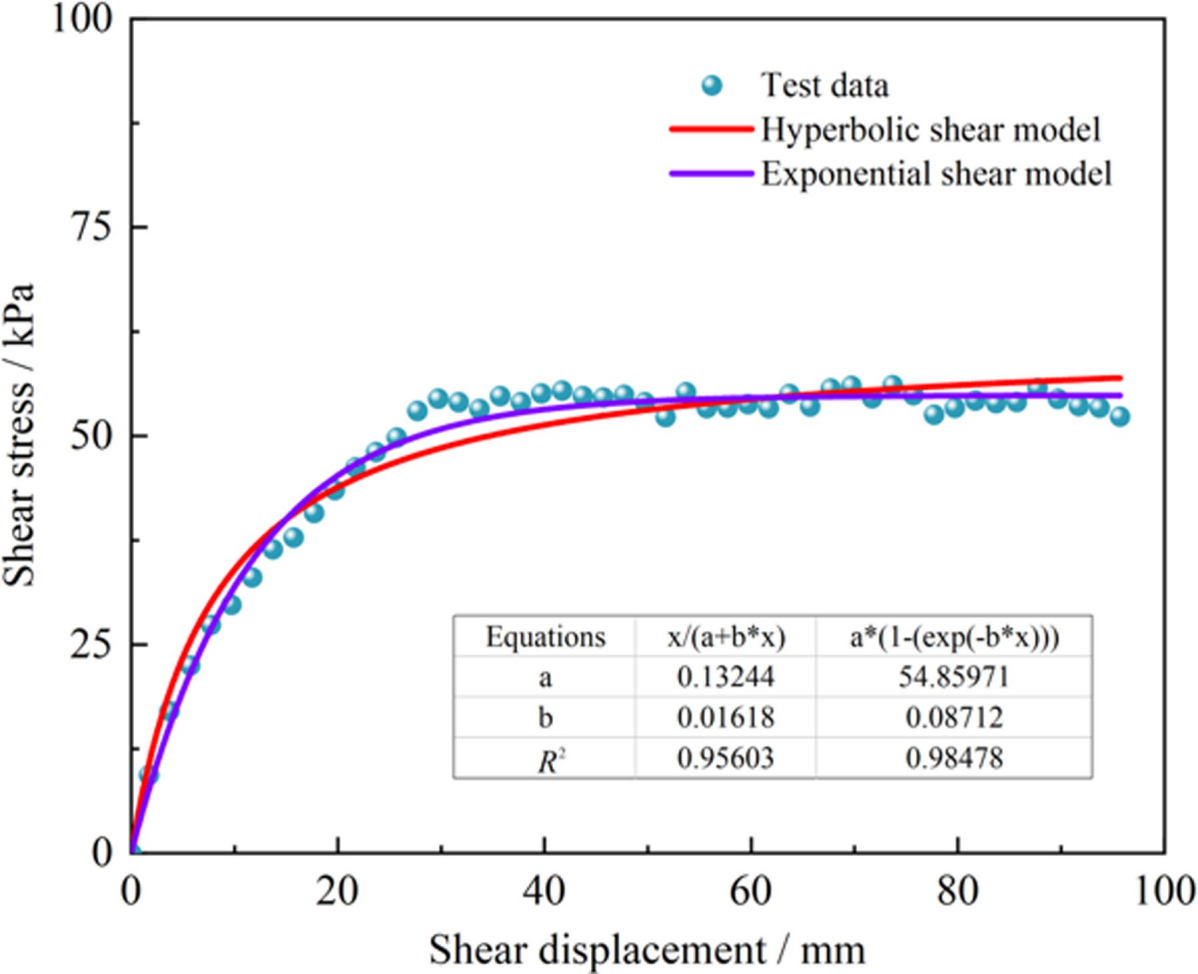
Comparison of traditional shear models.

The shear stress-shear displacement relationship curves obtained by fitting the shear test results using these two models are depicted in Fig 8. The figure indicates that the fitting of test data using the exponential and hyperbolic models is relatively effective, with correlation coefficients greater than 0.95. The exponential model fitting has a slightly larger correlation coefficient. However, a common limitation of both models is the absence of a peak after model fitting. Usually, the asymptote equation is used to estimate the peak shear stress, but these estimates do not accurately describe the material’s low shear performance. Furthermore, a minor softening phenomenon is observed post-peak in the direct shear test.

### Establishment of fractional derivative shear model

From a physics standpoint, the partial derivative of shear stress with respect to shear displacement is understood as a conventional relaxation equation [28], as depicted in Eq (7):

$$\frac{\partial \tau (x)}{\partial x}=\frac{K_{si}}{\tau_{m}}\left[\tau_{m}-\tau (x)\right]$$ (7)

Eq (7) illustrates that the shear stiffness decreases from *K*_*si*_ exponent to 0. To consider the time effect of soil-rock mixture’s shear deformation, we use the fractional relaxation equation instead of the ordinary first-order relaxation equation, yielding Eq (8):

$$\frac{\partial^{\lambda }\tau (\text{x})}{\partial x^{\lambda }}=\frac{K_{si}}{\tau_{m}}\left[\tau_{m}-\tau (x)\right]$$ (8)

In this equation, $\frac{\partial^{\lambda }\tau (\text{x})}{\partial x^{\lambda }}$ represents the fractional derivative, *λ* is the fractional order. Numerous definitions exist for the fractional derivative; in this paper, we have used the Caputo theory [29], as defined in Eq (9):

$$\frac{\partial^{\lambda }\tau (x)}{\partial x^{\lambda }}=\frac{1}{\Gamma (n-\lambda )}\int_{0}^{x}{\left(x-s\right)}^{n-\lambda -1}f^{\left(\lambda \right)}(s)\text{d}s$$ (9)

In this equation, when *λ* > 0 and *n*– 1 < *λ* < *n* are satisfied, Γ(*n*– *λ*) represents the Gamma function, defined in Eq (10):

$$\Gamma (n-\lambda )=\int_{0}^{\infty }{\text{e}}^{-t}t^{n-\lambda -1}dt$$ (10)

Performing a Laplace transform on Eq (8) results in Eq (11):

$$\tau (x)=\tau_{m}E_{\lambda }\left\{-\frac{K_{si}}{\tau_{m}}x^{\lambda }\right\}$$ (11)

In Eq (11), *E*_*λ*_(*z*) is a one-parameter Mittag-Leffler function, symbolizing the generalized extension of the exponential function in the sense of fractional calculus, defined in Eq (12):

$$E_{\lambda }(z)=\sum_{k=0}^{\infty }\frac{z^{k}}{\Gamma (\lambda k+1)}$$ (12)

We can similarly derive the fractional derivative shear model equation under the Mittag-Leffler function, as shown in Eq (13):

$$\tau (x)=\tau_{m}\cdot \left[1-\exp(-\frac{K_{\text{si}}}{\tau_{m}}\cdot x^{\lambda })\right]$$ (13)

### Characteristics of fractional derivative shear model

The first feature: over the origin: when *x* = 0 is satisfied, *τ*(*x*) = 0.

The second feature: there is residual shear stress: when *x* approaches infinity, *τ*(*x*) = *τ*_*m*_.

The third feature: there is a softening phenomenon after the peak. We obtain shear stiffness *K*, from the first derivative of Eq (10), as given in Eq (14):

$$K=\frac{d\tau }{dx}=K_{si}\cdot \lambda \cdot x^{\lambda -1}\exp(-\frac{K_{si}}{\tau_{m}}\cdot x^{\lambda })$$ (14)

Taking the second derivative of Eq (14) gives Eq (15):

$$\frac{d^{{}^{2}}\tau }{dx^{2}}=K_{si}\cdot \lambda \cdot \exp(-\frac{K_{si}}{\tau_{m}}\cdot x^{\lambda })\left[(\lambda -1)\cdot x^{\lambda -2}-\frac{K_{si}}{\tau_{m}}\cdot \lambda \cdot x^{2(\lambda -1)}\right]$$ (15)

Setting Eq (15) equal to 0 gives Eq (16):

$$x=\left\{\begin{array}{c} 0\\ {}^{{\left(\frac{\tau_{m}(\lambda -1)}{K_{si}\cdot \lambda }\right)}^{\frac{1}{\lambda }}}\\ \infty \end{array}\right.\Rightarrow K=\left\{\begin{array}{l} >0,x\in (0,x_{0})\\ =0,x=x_{0}\\ <0,x\in (x_{0},\infty ) \end{array}\right.$$ (16)

Eq (16) demonstrates that as shear displacement value increases, the shear stress of this shear model increases monotonically to a maximum value, then decreases to a stable value, matching the strain-softening phenomenon of soil-rock mixture after reaching peak shear stress.

As defined in Eq (17):

$$x_{0}={\left(\frac{\tau_{m}(\lambda -1)}{K_{si}\cdot \lambda }\right)}^{\frac{1}{\lambda }}$$ (17)

We can substitute Eq (16) into Eq (12) to obtain the peak shear stress *τ*_max_, as per Eq (18):

$$\tau_{\text{max}}=\tau_{m}[1-\exp(\frac{\left(1-\lambda \right)}{\lambda })]$$ (18)

Substituting Eq (16) into Eq (13) gives the peak shear stiffness *K*, as per Eq (19):

$$K=\tau_{m}\cdot {\left(\lambda -1\right)}^{\frac{1-\lambda }{\lambda }}{\left(\frac{K_{si}}{\tau_{m}}\cdot \lambda \right)}^{\frac{1}{\lambda }}\cdot \exp(\frac{1-\lambda }{\lambda })$$ (19)

From these characteristics, it is clear that the fractional derivative shear model can predict the entire shear failure process of a soil-rock mixture more accurately.

### Verification of fractional derivative shear model

**Fig 9:**
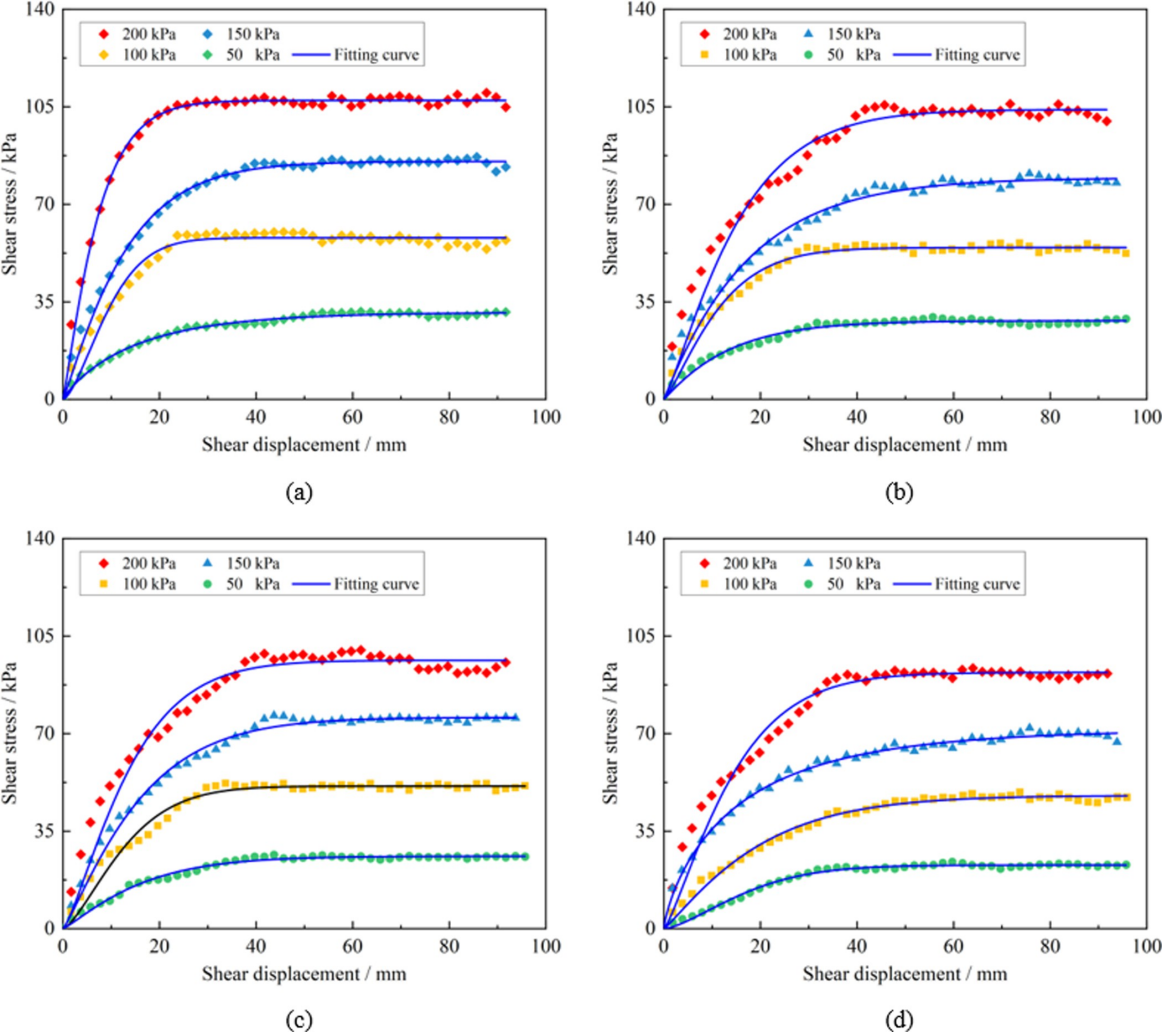
Model fitting curve under different moisture content. (a) Water content 4.2%, (b) Water content 8.4%, (c) Water content 16.8%, (d) Water content 33.6%.

The fractional derivative shear model has been used to fit the shear test data of a soil-rock mixture with varying water content, resulting in a fitting curve that encapsulates 16 sets of test data, as shown in Fig 9. In the figure, *R*^2^ represents the correlation coefficient, and *λ* denotes the fractional order. The specifics of the test data, in comparison to the fitting data from the fractional derivative shear model, are detailed in Table 5. A relative error of less than 5% is observed between the peak shear stress derived from the model and the experimental peak shear stress. The correlation coefficient *R*^2^ is greater than 0.99, nearing 1, suggesting that the model error is quite small. From the data presented in Table 5, it is evident that the *λ* values obtained in this test range between 0.75 and 1.60. When the *λ* value equals 1, the fractional derivative shear model simplifies into an exponential shear model. Overall, it is clear that the model fitting results proposed in this study are not only better but also more accurate. They effectively reflect the entire process of shear deformation in soil-rock mixture.

**Table 5:** Comparison of test data and shear model fitting data.

Water content /(%)	Overburden load/kPa	Test peak shear stress/kPa	Fitting peak shear stress/kPa	Relative error/(%)	Initial shear stiffness *K*_3_/(kPa/mm)	*λ*	Correlation coefficient
4.2	50	31.49	31.23	0.83	2.68	0.90	0.9993
	100	60.05	57.98	3.45	1.49	1.54	0.9968
	150	86.99	85.39	1.84	4.84	1.12	0.9991
	200	109.99	107.28	2.46	10.47	1.15	0.9990
8.4	50	29.50	28.22	4.34	1.87	1.04	0.9990
	100	56.08	54.46	2.89	2.31	1.27	0.9985
	150	81.01	79.49	1.88	4.26	1.02	0.9980
	200	106.00	103.98	1.91	3.71	1.20	0.9965
16.8	50	26.50	26.00	1.89	0.99	1.17	0.9994
	100	52.05	51.16	1.71	1.15	1.43	0.9984
	150	76.51	75.81	0.91	2.85	1.17	0.9984
	200	100.01	96.28	3.73	2.94	1.29	0.9948
33.6	50	23.98	22.81	4.88	0.28	1.49	0.9996
	100	49.00	47.81	2.43	1.59	1.14	0.9989
	150	72.01	71.55	0.64	8.31	0.78	0.9988
	200	93.51	91.94	1.68	2.96	1.26	0.9976

## Discussion and conclusions

### Discussion

Soil-rock mixture is commonly used in various construction projects, including slope, mining, and building foundation engineering, serving as a filler. The mechanical properties of this material can be rather complex due to the significant variation in particle sizes. Therefore, many researchers have dedicated their work to studying the mechanical properties of soil-rock mixtures, with primary focuses on factors such as block stone content, strength variation, shear models, lithology, and soil properties. Given the increasing applications of soil-rock mixtures, future studies on this topic are expected to be more detailed, in-depth, and varied in methodologies. In this paper, the impacts of normal stress, water content, and particle characteristics on the mechanical properties of soil-rock mixtures have been analyzed through large-scale direct shear tests. From these analyses, a fractional derivative shear model has been developed. However, it should be noted that soil-rock mixtures with structural interlayers possess distinct characteristics. It is essential to further investigate how the composition of the soil-rock mixed interlayer, particularly the properties of gravel and cements, affects its shear performance. From a microscopic to a macroscopic perspective, a damage model for rock masses containing soil-rock mixed interlayers needs to be established.

### Conclusions

1. Moisture content is a pivotal factor. As moisture content increases, the peak shear stress of soil-rock mixture gradually decreases, exhibiting strain-softening phenomenon after reaching peak shear stress. Specifically, when the water content increases from 4.2% to 33.6%, the cohesion of the soil-rock mixture decreases by 73.47%, and the internal friction angle decreases by 11.11%. Nonlinear fitting shows that the cohesion and internal friction angle of the soil-rock mixture decrease as a power function of the increase in water content.
2. Under the same mass fraction of fine particles, a higher overburden pressure increases the compactness and the corresponding shear strength of the soil-rock mixture. Conversely, under the same overlying pressure, an increase in the mass fraction of fine particles weakens the overall shear capacity of the soil-rock mixture. When the mass fraction of fine particles increases from 8.56% to 34.24%, the cohesion of soil-rock mixture decreases by 39.98%, and the internal friction angle decreases by 22.57%.
3. Through data fitting, a fractional derivative shear model was established by introducing the fractional order. This model accounts for the softening phenomenon observed after the peak shear stress and can better simulate the entire shear deformation process of the soil-rock mixture. A comparison and analysis of the shear test results of the soil-rock mixture under different water content conditions showed that the relative error between the peak shear stress fitted by the model and the peak shear stress of the test is less than 5%, and the correlation coefficient *R*^2^ is greater than 0.99. This small model error supports the rationality of the model.
